# Infectious Disease Modeling with Socio-Viral Behavioral Aspects—Lessons Learned from the Spread of SARS-CoV-2 in a University

**DOI:** 10.3390/tropicalmed7100289

**Published:** 2022-10-09

**Authors:** Nuning Nuraini, Kamal Khairudin Sukandar, Maria Yulita Trida Tahu, Ernawati Arifin Giri-Rachman, Anggraini Barlian, Sri Harjati Suhardi, Udjianna Sekteria Pasaribu, Sonny Yuliar, Diky Mudhakir, Herto Dwi Ariesyady, Dian Rosleine, Iyan Sofyan, Widjaja Martokusumo

**Affiliations:** 1 Department of Mathematics, Faculty of Mathematics and Natural Sciences, Institut Teknologi Bandung, Bandung 40132, Indonesia; 2 School of Life Science and Technology, Institut Teknologi Bandung, Bandung 40132, Indonesia; 3 Center of Public Policy and Governance, Institut Teknologi Bandung, Bandung 40132, Indonesia; 4 School of Pharmacy, Institut Teknologi Bandung, Bandung 40132, Indonesia; 5 Faculty of Civil and Environmental Engineering, Institut Teknologi Bandung, Bandung 40132, Indonesia; 6 General Administration and Information Bureau, Institut Teknologi Bandung, Bandung 40132, Indonesia; 7 School of Architecture, Planning and Policy Development, Institut Teknologi Bandung, Bandung 40132, Indonesia

**Keywords:** SIR model, socio-behavioral aspects, interaction distance, school reopening strategy

## Abstract

When it comes to understanding the spread of COVID-19, recent studies have shown that pathogens can be transmitted in two ways: direct contact and airborne pathogens. While the former is strongly related to the distancing behavior of people in society, the latter are associated with the length of the period in which the airborne pathogens remain active. Considering those facts, we constructed a compartmental model with a time-dependent transmission rate that incorporates the two sources of infection. This paper provides an analytical and numerical study of the model that validates trivial insights related to disease spread in a responsive society. As a case study, we applied the model to the COVID-19 spread data from a university environment, namely, the Institut Teknologi Bandung, Indonesia, during its early reopening stage, with a constant number of students. The results show a significant fit between the rendered model and the recorded cases of infections. The extrapolated trajectories indicate the resurgence of cases as students’ interaction distance approaches its natural level. The assessment of several strategies is undertaken in this study in order to assist with the school reopening process.

## 1. Introduction

In epidemiology, compartmental models are general modeling techniques used to understand the spread of disease, and they commonly consider three variables: S for those who are susceptible, I for those who are infected, and R for individuals who have recovered. Variations of the generic SIR model are available: the SIS model accommodates temporal immunity [1], the SEIR model best represents the spread of disease with a significant latency period [2], and there are even combinations of the two [3]. The convenience of compartmental models in respect of adding more variables has resulted in their being widely used in infectious disease modeling [4]. Besides providing each state’s estimated figure, this approach can also provide the reproductive ratio, which represents the expected number of secondary cases generated by one primary case [5,6,7]. In most of the constructed models, the reproductive ratio acts as a crucial threshold; above one indicates endemic, while below one indicates disease-free [8]. This is crucial for policymakers when regulating whether or not to ease restrictions amid disease spread.

However, generic compartmental models are sometimes based on assumptions that are not necessarily relevant; the population is considered closed in SIR models, whereas complete isolation was not followed in most regions, making them vulnerable to changes in the neighboring communities [9]. Another assumption that is commonly used in a generic model is that transmission and recovery rates are assumed to remain constant over time. Such a scenario will best represent disease that spreads in a population with no response to current disease prevalence, meaning that a high or low number of recorded cases will not affect the average socio-behavior of the population. The simplest case to consider is a disease spread within a closed population of sheep in a field [10]. When it comes to a human population, people’s psychological behavior causes them to reduce their interaction intensity as the declared number of cases increases, which ought to vary the viral transmissibility [11]. Moreover, setting constant rates of transmission and recovery results in a high number of projected infected cases once the model is applied to a vast and highly populated community; this could be at the scale of entire nations [12]. According to recent studies, SIR-based predictions using early data for COVID-19 cases have shown an enormous figure for predicted cases, with the peak reaching up to 15–30% of the total population [13,14]. Nevertheless, an absence of the psychological behavior of the population could overestimate the prediction figure [15].

According to recent studies, there are so many studies that discuss the spread of the COVID-19 disease. Researchers developed various models and approaches from all over the world [16,17]. However, in this paper, we will discuss two major sources of transmission in some infectious diseases: direct contact and airborne transmission. In respect of the former, it is quite obvious that human-to-human transmission is mainly caused by direct contact such as talking at a close distance. The smaller the average interaction distance of people within a population, the greater the chance for pathogens to spread. By incorporating the effect of human psychological behaviors, it is natural to expect an increase in the average interaction distance given a high disease prevalence in a specific population, which will lead to a reduction in viral transmissibility. However, the latter source of transmission opens up possibilities for infections induced by the presence of airborne pathogens. This method of transmission is found in the spread of TB [18] and SARS-CoV-2 [19]. Although airborne pathogens can infect susceptible individuals, some studies have shown that most airborne pathogens can only last for a certain period. *Mycobacterium tuberculosis*, which attacks lungs and causes TB, can stay in the air for several hours depending on the environment [20], and SARS-CoV-2 can only last for hours in the air but can survive for up to a week on plastic [21]. In disease modeling, taking airborne pathogens into account is crucial, especially for those that have a significant period of viral survivability in the air.

The incorporation of the psychological behavior of society into responses to disease prevalence has been introduced in several works, such as Hua-Li et al. [11] and Oluyori et al. [22]. In practice, the authors define saturated transmission rates that are dependent on the figure of disease prevalence. The transmission rate is expected to increase for a low disease prevalence and start decreasing once the prevalence exceeds its critical point [11]. In 2021, Cabrera et al. [23] introduced a compartmental model that incorporates a socio-behavioral aspect in a slightly different way; they introduced the interaction distance to measure societal behaviors in response to disease prevalence. Hence, the nonlinear transmission rate integrates the interaction distances. However, the effect of airborne pathogens is rarely incorporated. One study conducted by Bazant and Bush in 2021 [24] demonstrates the significant effect of airborne transmissions on society regarding activities. Although airborne pathogens, especially SARS-CoV-2, can only last for hours, indoor transmission is crucial for infectious disease modeling, especially for school or office environments involving many indoor activities.

In this study, we constructed an *SIR*-based mathematical system that accommodates the two major causes of infection: direct contact and airborne transmission. The former source of infection, representing the socio-behavioral aspect, is based on the measure of the interaction distance of people in society. In 2021, Cabrera et al. [23] proposed adding a new variable that determines the interaction distance over time. The closer the interaction distance, the higher the chance of disease spread. The latter cause of infection, which represents viral characteristics, is incorporated by defining another variable that solely represents the concentration of pathogens in the air over time. We expect that the longer the pathogens can last in the air, the higher the concentrations over time, which leads to a higher chance of disease spread. Hence, the newly added variables will govern the transmission rate that eventually depends on the socio-viral behavioral aspects. In the analysis of the constructed model, we provide numerical results in respect of infections under different socio-viral behavioral aspects. The model performs well in depicting the spread of disease in societies under different rates of response, different rates of resistance to adopting new habits, and under different characteristics of the concerned diseases. As a case study, we applied the constructed models to the SARS-CoV-2 spread data that were collected in a university environment (Institut Teknologi Bandung College) in January 2022. The choice to use data from a university was made to ensure homogeneous socio-behavioral aspects for the whole society; no demographic is taken into account due to the homogeneity assumption [25,26,27]. The small scale of a university environment also ensures the involvement of pathogens in the air; the larger the scale of the observation, the smaller the effect of pathogens in the air. Lastly, we utilized the extrapolated figures to assess some strategic action plans related to SARS-CoV-2 infections in educational environments; school reopening schemes and vaccination implementation [28].

## 2. Context

Humans are mobile creatures who move in their part of an environment; they may meet an acquaintance or not. When the former scenario happens, they will likely move closer to reaching out to that acquaintance [29]. This phenomenon exemplifies the importance of interpersonal space (IPS) and peripersonal space (PPS) in which humans can perform body–environment interactions [30]. Although the dimensions of IPS and PPS include all directions, previous studies have only focused on a specific distance, i.e., the distance from the front of the person [31]. When it comes to understanding infectious diseases, the front-directed PPS is essential since most diseases, including SARS-CoV-2, are transmitted via the front parts of the human body. One unit that measures the intensity of PPS contact is the interaction distance, in which the closer the distance, the more intense the contact, which leads to an increase in the risk of disease transmission [32]. According to Sorokowska et al. [33], the preferred interpersonal distance of humans differs between different types of social relations (strangers, acquaintances, and partners). Table 1 provides a global comparison in respect of interaction distance.

Other than the interaction distance that causes direct transmissions, airborne transmission of some diseases is now widely recognized, especially for the spread of COVID-19 [34,35]. This approach accounts for the plausibility of infections caused by pathogen-bearing aerosols that are fine enough to be continuously mixed through an indoor space. Every infected individual present will contribute to the production of droplets containing the virus. Bazant and Bush [24], in their COVID-19 study, estimated the concentration of pathogens produced by a single infected individual in a well-mixed room for every breath, and for whispering and talking indoors.

However, other studies have shown that pathogens can remain active on other media, such as copper, cardboard, and plastic [21], for a certain period. Hence, other than significant airborne transmission indoors, pathogens that are attached to other media can also infect susceptible individuals. A study by Doremalen et al. provides the estimated critical periods of SARS-CoV (1 and 2) before they become inactive; these are given in Table 2. The estimations show that SARS-CoV can last up to 12 h in the air but can last longer on other media. This fact should indicate the importance of airborne pathogens and their attachment to other media in respect of understanding viral transmission. In this study we construct a mathematical model that incorporates both socio-behavioral and airborne pathogen effects.

## 3. Proposed Model

**Figure 1:**
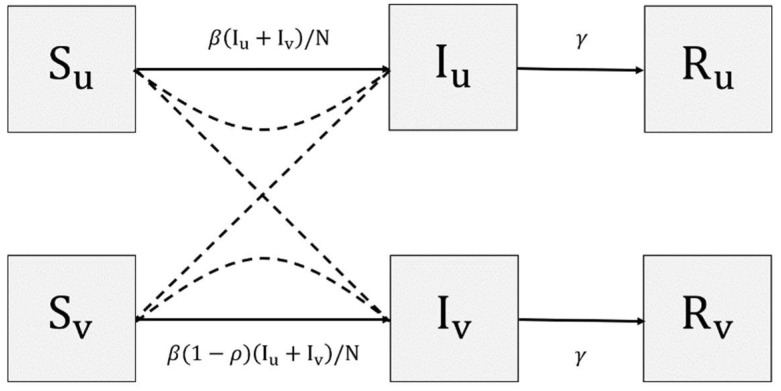
State-flow diagram of ${\left\{SIR\right\}}_{uv}$. Solid lines represent direct flow, while dashed lines represent interactions of states.

In this study we used a generic model, but we separated those who had and had not received vaccines. This modification was based on the fact that the presence of immune titer in the human body can significantly prevent people from becoming infected, offering up to 90% protection [36]. Hence, there are three main state variables: susceptible (S), currently infected individuals (I), and removed individuals (R), with the total of six state variables created by adding subscripts v and u to each of the main states, representing the categories of being vaccinated and not, respectively. As shown in Figure 1, new infected individuals are generated from both $S_{u}$ and $S_{v},$ caused by a direct interaction between susceptible and infectious individuals. After a specific period of infections, infected individuals will enter R, which represents being immune or deceased. We assume that there is no demographic change, which implies a constant population size: $N_{u}=S_{u}\left(t\right)+I\left(t\right)+R_{u}\left(t\right)$ and $N_{v}=S_{v}\left(t\right)+I_{v}\left(t\right)+R_{v}\left(t\right)$, for t≥0, and $N=N_{u}+N_{v}$ with a constant proportion of vaccinated and unvaccinated individuals. The model also assumes no significant difference in the recovery rates of vaccinated and unvaccinated individuals.

As shown in Figure 1, there are three parameters involved: transmission rate (β), recovery rate (γ), and vaccine effectiveness (ρ). The last two parameters are observable, i.e., their values can be measured and estimated using relevant information. Vaccine effectiveness, which ranges from 0 to 1, represents the protection induced by the vaccine. The higher this value, the lower the chance of people becoming infected once they interact with infectious individuals. Limited to the COVID-19 vaccine, the vaccine efficacy should vary depending on the manufacturer and COVID-19 variants [36]. The value of γ represents the rate of recovery, which governs the speed of transition from I to R. To make this realistic, $\gamma^{-1}$ can be considered as the average infection period. In contrast, the rate of transmission β is unclear in terms of its physical representation; it summarizes all factors that produce infections. Hence, the value of *β* is considered unobservable. To incorporate the two major causes of infection as mentioned in Section 2, we added two additional lines to the system that represent the dynamics of the interaction distance *D* and the pathogen concentrations V. The final two variables dictate the dynamics of β resulting in the transmission rate that depends on the socio-viral behavioral aspect. A mathematical representation of the constructed model is given in the following form:

(1) $$\left\{ \begin{array}{c} S_{u}^{\prime }=-\beta \left(I_{u}+I_{v}\right)S_{u}/N\\ I_{u}^{\prime }=\beta \left(I_{u}+I_{v}\right)S_{u}/N-\gamma I_{u}\\ R_{u}^{\prime }=\gamma I_{u}\\ S_{v}^{\prime }=-\beta (1-\rho )\left(I_{u}+I_{v}\right)S_{u}/N\\ I_{v}^{\prime }=\beta (1-\rho )\left(I_{u}+I_{v}\right)S_{u}/N-\gamma I_{v}\\ R_{v}^{\prime }=\gamma I_{u} \end{array}\right.$$

with a constant population size N. The other two additional variables are D (in meters) and V (in quanta/$m^{3}$), representing the average interaction distance and viral loads over time. The formulation of D was first introduced by Cabrera et al. [23] along with the definition of the natural distancing habit $D^{*}$ that could differ from one society to others—symbol $D^{*}$ denotes the average of natural distancing behavior of society. The complete additional lines are given in the following systems:

(2) $$\{\begin{array}{c} \;\;\;\;D^{\prime }=-\lambda_{1}\left(D-D^{*}\right)+\lambda_{2}\left(I_{u}+I_{v}\right)/N\\ \;\;\;\;V^{\prime }=\lambda_{3}\left(I_{u}+I_{v}\right)-\lambda_{4}V \end{array}$$

with non-negative initial conditions $\left\{S_{u}^{0},I_{u}^{0},R_{u}^{0},S_{v}^{0},I_{v}^{0},R_{v}^{0},D^{0},V^{0}\right\}$ that are evaluated at the initial point t=0. It is natural to assume that $I_{u}^{0}=\left(1-\alpha \right)I^{0}$ and $I_{v}^{0}=\alpha I^{0}$, for $I^{0}=I_{u}^{0}+I_{v}^{0}$, with α (in percent relative to the population size) representing the vaccine coverage. The addition of the two variables involves another four parameters. On one hand, the value of $\lambda_{2}$ (distance/time) represents how quickly people react to the current disease prevalence, i.e., the so-called rate of social response. By neglecting the first term, there are two scenarios that increase the interaction distance D: high values of the rate of response $\lambda_{2}$ or the disease prevalence I. Interestingly, setting $\lambda_{2}$ equals zero will lead to a situation where a society pays no attention to the current disease spread. Such a scenario drives the society to resort to their natural interaction distance D for $\lambda_{1}\neq 0$. On the other hand, the rate $\lambda_{1}$ (1/time) measures the rate of resistance in society, per distance unit, to changing distancing behavior. It represents how quickly individuals return to their natural interaction distance $D^{*}$ or their natural distancing habits. This rate is strongly related to the distancing culture. When we set a high value of $\lambda_{1}$, this results in a situation in which the society has a strong culture embedded, making it resistant to changes in behavior amid the current pandemic. In this study, we restrict the plausibility of $\lambda_{1}=0$ since we assume that every society has its own resistance in changing habits. When the disease prevalence approaches zero, then $D^{\prime }$ approaches $-\lambda_{1}\left(D-D^{*}\right),$ which leads to the convergence of D to $D^{*}$ regardless of the initial condition $D^{0}$. More detailed formal analysis of System (1) and (2) are given in Appendix A. 

While the first equation of System (2) portrays the socio-behavioral aspects, the second equation portrays the concentration of the pathogens. The rate $\lambda_{3}$ (quanta/(time $m^{3}\cdot \mathrm{person}))$ denotes the average concentration of viral/pathogens emitted by one infected individual per unit time. Face coverings and the practice of other social and respiratory etiquette will likely reduce the value of $\lambda_{3}$ and hence reduce the number of pathogens emitted into the air. The discharged microbes will remain suspended in the air in dust particles, respiratory particles, and water droplets [37]. However, pathogens will not last forever in the air (or other media); they will decay due to natural and human intervention. On the other hand, parameter $\lambda_{4}$ (1/time) denotes the removal rate of viral quanta in the air. A higher intervention of humans in the community, including through air filtering and periodical sanitation, can increase $\lambda_{4}$ and hence allow more microbes to decay or be inactive [38]. However, in most cases, $\lambda_{4}$ will only account for the natural effect of pathogen removal (subject to ambient temperature, humidity [21,39], and sunlight [40]), while human intervention can be represented by another functional term added to the dynamic of V [41]. Eventually, $\lambda_{1},\;\lambda_{2,}$ and $\lambda_{3}$ represent the socio-behavioral aspects in society while $\lambda_{4}$ represents the characteristics of the pathogens.

(3) $$D\left(t\right)=D^{*}+\left(D^{0}-D^{*}\right)e^{-\lambda_{1}t}+\frac{\lambda_{2}}{N}\int_{0}^{t}I\left(s\right)e^{-\lambda_{1}\left(t-s\right)}ds$$

(4) $$V\left(t\right)=V^{0}e^{-\lambda_{4}t}+\lambda_{3}\int_{0}^{t}I\left(s\right)e^{-\lambda_{4}\left(t-s\right)}ds$$

Since the model adopts a uni-flow, then there exists τ such as I(t)<ε, t>τ, for every ε>0. In terms of epidemiology, the virus will always be eradicated to zero for large values of *t* since people will accumulate in the removed compartments. For the dynamics of D, the second and third terms approach zero as t approaches infinity, leaving only the first term that converges to $D^{*}$. However, the presence of V is strongly related to the presence of infectious individuals, who will vanish once the disease vanishes, no matter how large the initial condition. It should be noted that the proposed models do not consider reinfection or susceptible newborns. Hence, multiple disease outbreaks (if any) are expected to be driven by the change in interaction distance in society.

### 3.1. Observability of Socio-Behavioral Parameters

As discussed in the previous section, the model has 3 parameters that are related to the socio-behavioral aspects of society: $\lambda_{1}$, $\lambda_{2},$ and $\lambda_{3}$. It is clear from its definition that $\lambda_{3}$ is observable and that its value follows the estimations of the pathogen concentration per person per $m^{3}$. Bazant and Bush [24] and Miller et al. [42] provided estimated concentrations for several expiratory activities. Calibrated normal speaking activity is estimated to produce 72 infections quanta/$m^{3}$ while superspreading activity can contribute up to 970 infections quanta/m_3_. However, the first two socio-behavioral parameters are not observable, i.e., the rate of social resistance $\lambda_{1}$ is not something that we can determine from the field. It combines all aspects that inhibit society in the change of behaviors.

The rate of social response, denoted by $\lambda_{2},$ has a dimension of meters per unit of time. In the absence of $\lambda_{1}$, the formula of $D^{\prime }$ reduces to only $D^{\prime }=\lambda_{2}\left(I/N\right)$, with $I=I_{u}+I_{v}$. When *I* = *N*, then $D^{\prime }=\lambda_{2}$, which is interpreted as the interaction distance increasing at the rate of $\lambda_{2}$ meters per unit time when the whole population is infected. Taking another scenario, I=1 person results in $D^{\prime }=\lambda_{2}/N$, which is considered as the $\lambda_{2}/N$ increment of the interaction distance per unit of time when the society contains 1 infected individual. Henceforth, $\lambda_{2}$ is related to the quantity of the change in D for a certain disease prevalence. To understand this parameter more, let us take the solution of $D^{\prime }=\lambda_{2}\left(I/N\right);\;D\left(t\right)-D^{0}=\lambda_{2}\int_{0}^{t}\left(I\left(s\right)/N\right)ds$. By taking $D^{0}=D^{*}$, then $\lambda_{2}=\left(D\left(t\right)-D^{*}\right)/\int_{0}^{t}\left(I\left(s\right)/N\right)ds$. Expecting the presence of an average prevalence of $\bar{I}$ in the length of time $T_{2}$ will drive people in society to interact at the distance of $\bar{D}$, then $\lambda_{2}$ can be estimated using the following formula:

(5) $$\lambda_{2}=\frac{\left(\bar{D}-D^{*}\right)}{T_{2}\left(\frac{I}{N}\right)}$$

Note that $\bar{I}/N$ represents the percentage of infections in society, i.e., the so-called point prevalence, denoted by a%. Therefore, by knowing that the society is practicing distancing habits of $D=\bar{D}$ once the point prevalence is roughly a%, we can estimate the expected value of *λ*_2_ as the rate of social response amid the disease spread. Henceforth, $\lambda_{2}=\left(\bar{D}-D^{*}\right)/\left(aT_{2}\right)$.

We can also consider the dynamics of D in the given system. When $\left(I_{u}+I_{v}\right)/N$ tends to zero, the effect of $\lambda_{2}$ is no longer significant; the whole second term will tend to zero, leaving $D^{\prime }=-\lambda_{1}\left(\bar{D}-D^{*}\right)$. This simple ODE has a unique solution of $D\left(t\right)=D^{*}+D^{0}e^{-\lambda_{1}t}$. The higher the value of $\lambda_{1}$, the faster the dynamics of D to approach $D^{*}$. It is easy to prove that $\underset{t\to \infty }{\lim}D\left(t\right)=D^{*}$, regardless of the value of $D^{0}$. Hence, for an arbitrary small value ε>0, there exists a value of $T_{1}$ that satisfies the following condition.

(6) $$\left|D\left(t\right)-D^{*}\right|<\varepsilon \;\mathrm{for}\;t>T_{1}↔\frac{\left|D\left(t\right)-D^{*}\right|}{D^{0}}<\frac{\varepsilon }{D^{0}}=\bar{\varepsilon }\;\mathrm{for}\;t>T_{1}$$

We can manipulate the solution of D(t) to reach $D^{*}+\bar{\varepsilon }\;\mathrm{in}\;t=T_{1}$ by adjusting the value of $\lambda_{1}$ as given by:

(7) $$D^{*}+\varepsilon =D^{*}+D^{0}e^{-\lambda_{1}t}\to \lambda_{1}=\frac{-\mathrm{In}\left(\frac{\varepsilon }{D^{0}}\right)}{T_{1}}=-\frac{\mathrm{In}\left(\bar{\varepsilon }\right)}{T_{1}}$$

**Figure 2:**
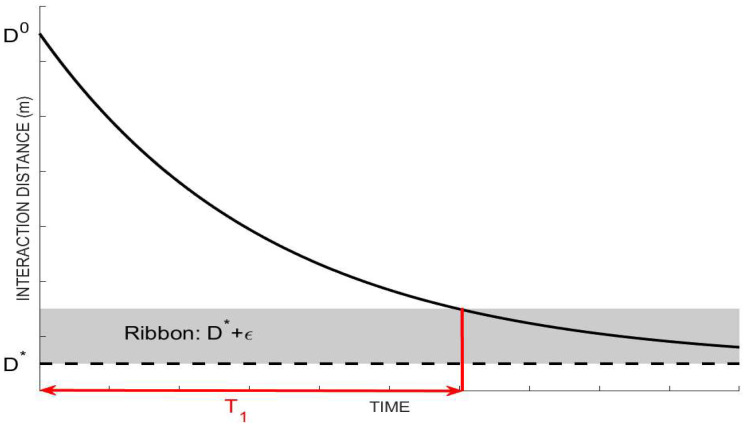
Illustration of rate of social resistance $\lambda_{1}$ by the given data for $T_{1}$.

Henceforth, the rate of social resistance $\lambda_{1}$ can be evaluated using the estimated time for society to return to their natural interaction distance in the absence of disease spread, denoted by $T_{1}$; see Figure 2 for illustration. It should be noted that $\bar{\varepsilon }$ is an arbitrary small number ε>0 divided by $D^{0}$. According to Equation (7), $\lambda_{1}$ takes the log value of $\bar{\varepsilon },$ which will be sensitive to the choice of $\bar{\varepsilon }$. Hence, it is natural to assume the relative deviation from $D^{*}$ as $\bar{\varepsilon }=1\%,$ although the formula of $\lambda_{1}$ should clearly confirm that the value of $\lambda_{1}$ is dependent on the assumption.

### 3.2. Contact and Airborne-Based Transmission Rate

The rate of transmission is defined to be related to the interaction distance (D) and concentration of pathogens (V). In this study we accommodate two methods of transmission: contact-based and airborne-based transmission. Contact-based transmission is affected by the average interaction distance; the transmission rate decreases as the average interaction-distance increases, as people practice social-distancing. However, a high concentration of airborne pathogens contributes to an increase in the transmission rate.

(8) $$\beta \left(D,V\right)=\beta^{*}{\left(\frac{2D^{*}}{D^{*}+D}\right)}^{v}{\left(\frac{V+V^{*}}{V^{*}}\right)}^{w}$$

The definition of the contact-related transmission rate is adopted from [23], but we have added the effect of the current concentration of pathogens. The basic transmission rate $\beta^{*}\left(1/\mathrm{time}\right)$ is defined as constant, representing the basic probability of transmission per unit time. The second term (dimensionless) represents the effect of the average interaction-distance, which will decrease the overall β as D increases. The third term, however, represents how the concentration of pathogens affects the overall *β* value at which the risk of infection will rise as V increases. To keep the effect of V dimensionless, we divide V by the standard number of quanta exhaled by infectors per individual per $m^{3}$ per unit time. The adjuster levels of v and w are added to be fitted to the data, representing the strength of each source of infection in society.

### 3.3. Recovery Rate

**Figure 3:**
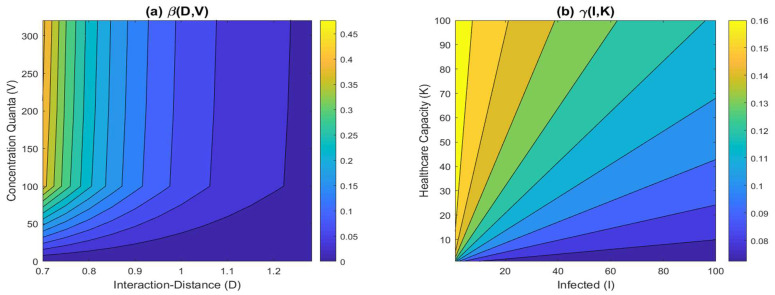
(**a**) Dependency of transmission rate β to D and V, by taking $\beta^{*}=0.444$, v=7.680, w=0.051 and $D^{*}=0.7$ m; (**b**) Effect of K and I to the rate of transmission by taking $\gamma_{1}=1/6$ (infection period of 6 days) and $\gamma_{0}=1/14$ (infection period of 14 days).

Recovery rates (1/time) denote the quantity representing how fast infected individuals recover from the disease and, hence, build their immunity [43]. For some infectious diseases, the absence of healthcare might cause a longer infection period [27,44,45], specifically for COVID-19. Not limited to this disease, we define the implicitly time-dependent recovery rate as follows:

(9) $$\gamma \left(I,K\right)=\gamma_{0}+\left(\gamma_{1}-\gamma_{0}\right)\frac{K}{I+K}$$

where I denotes the state variable for infectious individuals and K denotes the constant healthcare capacity (beds). In addition, $\gamma_{1}$ and $\gamma_{0}$ are both recovery rates but represent different situations: excessive beds and collapsing health systems. On one hand, when the number of beds is excessive, then each of the infected individuals receives proper treatment and this leads to a shorter period of infections [45]. In other words, γ(I, K) will achieve its maximum rate of recovery as *K* approaches infinity. Otherwise, γ(I, K) will converge to $\gamma_{0}$ as the number of burdens is higher relative to the healthcare services [11]. Hence, the former denotes the maximum recovery rate given the proper treatment, while the latter denotes the lowest recovery rate achieved by patients treating themselves in order to recover. Figure 3 depicts the functional parameters and their dependent variables. Figure 3 (left) illustrates the effect of the average interaction distance that results in higher values of β(D,V) as D approaches $D^{*}$. On the other hand, the rate of recovery follows Equation (9), which lessens the rate of the increase in the burden of cases down to $\gamma_{0}$. For the case with excessive healthcare capacities, the rate of recovery can be maximized up to $\gamma_{1}$, as shown in Figure 3 (right).

## 4. Numerical Results

In this study, the behavior of society that is being accommodated by the model is the rate of social resistance and social response. Socio-resistance rate, denoted by $\lambda_{1}$, represents the resistance of society to distancing their interactions due to the prevalence of people when I is not significantly zero. When the prevalence of people is close to zero, the resistance rate represents how fast the society moves back towards their natural interaction-distance $D^{*}$. In contrast, the rate of societal response represents the increase in interaction distancing per increase in point prevalence, which inhibits the disease spread when this value is high. In this section, we provide the number of infected individuals (per one thousand members of the population) for several values of $\lambda_{1}$ and $\lambda_{2}$.

### 4.1. Variations under Different Society Behaviors

The rate of social response is given in three scenarios (low, moderate, and high response), by taking values of $\lambda_{2}=0.20,0.53,$ and 0.87, respectively. These are based on the physical distancing campaign: (i) low social response drives people to physical distancing limited to $\bar{D}=1$ m only, (ii) moderate can reach $\bar{D}=1$.5 m, and (iii) high social response can reach up to 2 m. Table 3 shows the diverse approaches of countries in campaigning for physical distancing. We also set the rates of social resistance to $\lambda_{1}=0.15,\;0.07,$ and 0.05, which are based on $T_{1}=30,\;60,$ and 90 days, respectively. The ranges of $\lambda_{1}$ and $\lambda_{2}$ produced by Formulas (5) and (7) conform to those used in Cabrera et al. [23].

**Figure 4:**
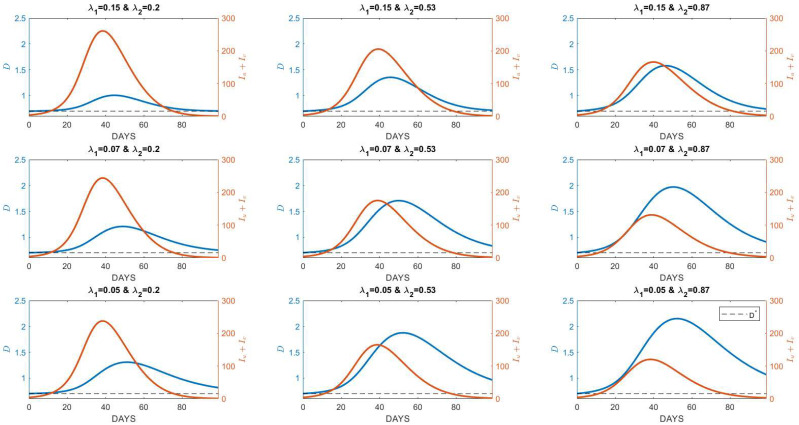
Numerical simulations for different values of the rate of resistance $\lambda_{1}$ and response to disease prevalence $\lambda_{2}$. Blue lines represent the average interaction distance, while the orange lines represent the burden of cases. The values of $\lambda_{1}$ and $\lambda_{2}$ are provided for three different values (low, moderate, high); $\lambda_{2}=0.20,\;0.53,\;0.87$ and $\lambda_{1}=0.05,\;0.07,\;0.15$. All figures were generated by choosing N=1000 and $D^{*}=0.7$ m and other parameters that evaluate the values of $R_{0}$ to exceed 1; $\rho =0.5,\;\gamma_{0}=1/14,$ $\lambda_{1}=1/6$.

Figure 4 provides the numerical simulations for $I_{u}+I_{v}$ and D for the different pairwise scenarios of $\lambda_{1}$ and $\lambda_{2}$. As expected, the value of D(t) will vary over time—increases as the disease prevalence increases. In all sub-figures, all dynamics for D(t) always start from $D^{*}$ as its natural distancing behavior when disease prevalence is around zero (no new cases recorded). However, as the disease prevalence rises, people in society build awareness to practice physical distancing which then increases the average distancing behavior D. As the new cases decrease to zero, it is natural that people in society return to their natural distancing $D^{*}.$

The sub-figure in the left upper corner depicts the simulation results for a society with a lower response yet a higher resistance rate. Such a scenario results in a higher peak of the burden of cases relative to other scenarios. This result shows that if the society does not have enough awareness about the disease’s prevalence, and has a strong resistance that inhibits the practice of physical distancing, the dynamics of D will be likely in around $D^{*},$ which results in a higher number of cases. Societies that campaign for close physical distancing (e.g., 1 m only), and have tendencies to always practice their natural habits, are likely represented by the left upper corner sub-figure. The figure situated at the center of the nine depicts simulations that apply to a society that has a considerably moderate level of resistance and response rate. The right lower corner depicts shows societies with a higher rate of response but a lower rate of resistance. Due to higher values of $\lambda_{2}$, every individual in the society moves further away relative to other scenarios and this results in a significant change in D relative to the value of $D^{*}$. When it comes to the figure of the burden of cases, this scenario estimates the lowest number of cases relative to other scenarios. Societies that practice physical distancing and have a tendency to keep practicing it in a longer period, even after the disease is no longer present, are best represented by this scenario, resulting in a lower burden of cases relative to other scenarios.

### 4.2. Variations under Different Pathogen Characteristics

**Figure 5:**
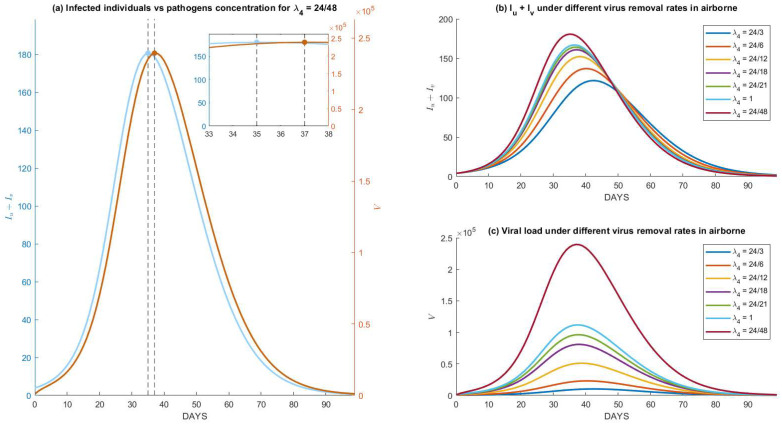
Numerical simulations of System (1) and (2) under different removal rates of airborne pathogens, that is implicated in the critical period of pathogens to remain active airborne: (**a**) comparison between the dynamics for infected individuals and viral load for a critical period of 48 h, which shows an exact lag of 48 h between the peak of infections and its viral loads, (**b**,**c**) dynamics for $I_{u}+I_{v}$ and its viral load under different critical periods of the airborne pathogen.

Different pathogens lead to different survivability periods in the air or other media. The longer the pathogens are active as airborne pathogens, the more they accumulate, which increases the risk of infections. Characteristics of the observed pathogens are governed by parameter $\lambda_{4}$. In System (2), the term $-\lambda_{4}V$ represents the concentration of pathogens per unit of time to become inactive. Figure 5 shows the dynamics of the disease prevalence $I_{u}+I_{v}$ under different periods of pathogens lasting in the air for the same parameters as used in Figure 4. In the lower-right picture, it is shown that pathogens that can last up to 48 h (red) can accumulate up to 300,000 quanta pathogens per $m^{3}$ and drive infections to as high as 23%. Figure 5a demonstrates how the dynamics of $I_{u}+I_{v}$ precede V on reaching a peak for exactly 2 days (48 h). It is natural to accept that the longer the period, the wider the gap between the occurrences of the two peaks. By setting a smaller period (higher $\lambda_{4})$, the dynamics of V decrease and so does $I_{u}+I_{v}$. Moreover, the peak of $I_{u}+I_{v}$ shifts to the right (see Figure 5b,c). More results on the model’s sensitivity analysis are provided in Appendix B and Appendix C.

## 5. Case Study: SARS-CoV-2 Spread in School

As mentioned in the previous section, the proposed model incorporates the socio-behavioral aspects of people in the society combined with the effect of airborne transmission. When it comes to socio-behavioral aspects, we included social resistance and social response amid the disease spread, which limit the scope of the implementation. At the scale of nations, people in society comprise all levels of education, culture, habits, or even wealth [48], which leads to a variety in perceptions when dealing with disease spread; some may have higher awareness but some may not. This fact challenges the modeler regarding how to estimate $\lambda_{1}$ and $\lambda_{2}$ that will accurately portray the society. Hence, we designed the model to be applied to the scale of an educational or office environment. It is natural to expect the homogeneity of socio-behaviors, even homogeneity in age, in schools or offices. These limitations also support the involvement of airborne transmission due to the indoor activity in schools or offices [24]. Henceforth, this section provides the applications of the proposed model to understand the disease spread in a university environment.

### 5.1. Dataset and Parameters’ Estimation

We collected the data in respect of the SARS-CoV-2 spread in a college environment, namely the Institut Teknologi Bandung (ITB), and data range from early January until late April 2022. The data comprise record daily cases, current active cases, and the total number of recovered individuals out of all enrolled students, lecturers, and college staff. Although students and staff do not stay at the college 24/7, it is reasonable to assume that they spend most of the time in the college environment. Here, we exclude the enrolled students that were infected in other cities due to the hybrid (online-offline) learning practice. The data are privately available at https://covidtrak.itb.ac.id/ (accessed on 1 April 2022), which is only accessible by ITB COVID-19 task-force members.

**Figure 6:**
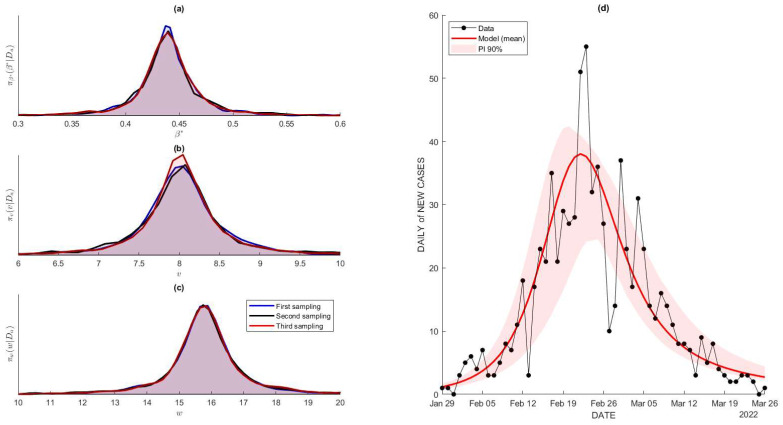
(**a**–**c**) Posterior distribution for $\beta^{*}$, w and w, estimated using MCMC method with prior of normal distributions: N(μ, σ), with μ being the estimated single point and σ being a higher value to acquire the possibility of achieving the global minima. We generated three independent samples to portray the posterior density to ensure its consistency. (**d**) The comparison between the data (daily new cases) and the model with its 90% prediction interval.

In terms of the parameter estimation, we only used data for the daily new cases from early January 2022 until late April 2022, which will be later denoted as $D_{a}$. Given in Table 4, System 204 and 210 involve 11 parameters, with only three of them being estimated by the integration of data $D_{a}$, namely $\beta^{*}$, v, and w, while other assumptions are as follows: the population size N equals 4000 (according to the report of the initial school reopening), the average vaccine efficacy ρ=0.37 for SinoVac [36], $\gamma_{0}=1/14,$ and $\gamma_{1}=1/6,$ which represent the rate of recovery under lack of and excessive healthcare, respectively. In order to obtain the estimations of $\beta^{*}$, v, and w, we used a Markov Chain Monte Carlo (MCMC) method to estimate the whole distribution. The complete Bayesian hierarchy for the MCMC method is provided in Appendix D. Figure 6 shows the estimated posterior distribution of $\beta^{*}$, *v*, and *w* that was implemented to the data that resemble the recorded daily new cases.

### 5.2. Projected Number of Cases

**Figure 7:**
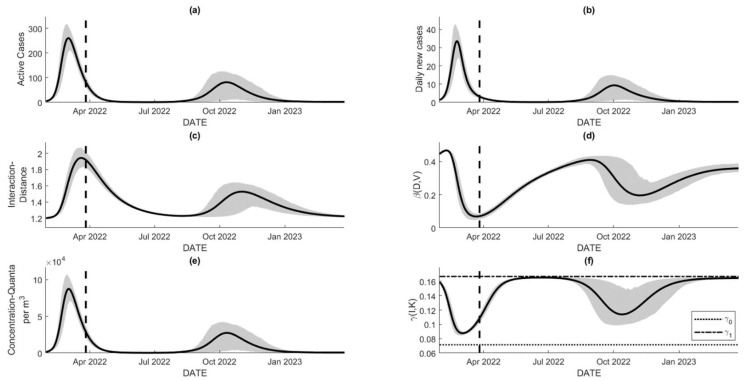
The extrapolated figures with the dashed line initiate the prediction window. The figures were evaluated using the estimated parameters obtained in the previous section, with the assumption of no significant change in parameters for the 365-day prediction intervals: (**a**,**b**) extrapolated number of active cases and daily new cases that levels off in around May to July 2022, but starts to increase on August and peaks on October 2022, (**c**) average interaction distance of society that will approach its natural distancing of $D^{*}=1.2$ m as the number of cases decreases, but increases when the case resurgence is identified, (**d**) time-dependent transmission rate that gradually increasing as D approaches $D^{*}$, (**e**) dynamic for viral load over time that resembles that for active cases, and (**f**) time-dependent recovery rate that its values are bounded by *γ*_0_ and $\gamma_{1}$.

Assuming no further changes in all parameters, the estimated posterior distribution of $\beta^{*}$, v, and w can be used to sample their values and generate the extrapolated trajectories for all states of the proposed model. Figure 7a and b show the projections of the disease prevalence in the university from early 2022 until mid-2023. Both consistently predict a significant decrease in the number of cases from May 2022, which implies a decrease in the average interaction distance D, approaching the social natural distancing $D^{*}$. Figure 7a–c clearly show that the figures are estimated with a relatively narrow prediction interval, which leads to high confidence in the results under the hold assumptions. As the average interaction distance D is around $D^{*}$, or, in other words, people in society behave as if there is no disease, the expected number of cases shown in Figure 7a,b increases in August 2022 and peaks in around October 2022, though the prediction interval is relatively wider compared to the previous period. These simulations show that the number of cases is expected to increase as D approaches $D^{*}$, without even considering reinfection due to immunity waning. 

Figure 7e depicts the dynamics of the pathogen concentrations in the observed area per $m^{3},$ which resembles the dynamics of the active cases over time. As stated in the previous section, the longer the pathogens can last in the air (or other media), the further the shift to the right relative to the dynamics of the active cases. In other words, the presence of $I_{u}+I_{v}$ contributes to the presence of pathogens that govern the rate of transmissibility. Figure 7d and f show the dynamics of β=β(D,V) and γ=γ(I,K). Although they are not directly dependent on time, they are time-dependent due to the dependency of D, V, and I to the unit of time. During the training time (initial time until the dashed lines), the rate of transmission β decreases due to the significant deviation of the average interaction distance relative to $D^{*}$. It is expected that the trajectories of β will increase during the prediction interval due to the decreasing values of D. For the rate of transmission, its value is always bounded within the $\gamma_{0}-\gamma_{1}$ ribbon. The rate is expected to approach the maximum value of γ as the burden of cases approaches zero; otherwise, it approaches the minimum γ. For Figure 7f, we set K=100, which represents the ability of the university hospital to accommodate only 100 patients at one time. This assumption causes a significant decrease in γ as the expected $I_{u}+I_{v}$ exceeds the value of K, depicting the ineffective health service as the burden exceeds its capacity.

### 5.3. Prospective Action Plans

Other than providing the extrapolated trajectories for all states, we are also interested in supplying numerical simulations related to prospective action plans for preventing the expected surge of COVID-19 in schools. This section provides the numerical assessment of three action plans: school reopening management, disinfection, and vaccine-related improvement.

#### 5.3.1. School Reopening Management

Although technologies support students in attending online classes, the practice of in-person classes is still preferable. This fact should be the main reason for the massive reopening of most Indonesian schools, regardless of the level of education. However, this should challenge the previous simulations due to a significant change in the number of individuals in a school as it is reopened. Henceforth, we provide numerical simulations of all states, more concerned with $I_{u}+I_{v}$, as the number of individuals in a school varies due to the school reopening. In practice, we assume that all individuals (students, lecturers, and staff) can be considered vulnerable to the disease. The higher the number of susceptible individuals, the more individuals can be infected. Hence, it is natural to analyze the effect of the increase in N on the dynamics of $I_{u}+I_{v}$.

Mathematically speaking, $N=S_{u}+I_{u}+R_{u}+S_{v}+I_{v}+R_{v}$, which implies that $N^{\prime }=S_{u}^{\prime }+I_{u}^{\prime }+R_{u}^{\prime }+S_{v}^{\prime }+I_{v}^{\prime }+R_{v}^{\prime }$. Substituting the derivatives of all states as stated in System (1), we have $N^{\prime }=0$, meaning that the population size remains unchanged. However, we modified the model to accommodate the change in the population size due to the school reopening. Since we assume that all new individuals enter compartments $S_{u}$ and $S_{v}$ (with the proportion governed by the vaccine coverage), we add recruitment terms f and g for $S_{u}$ and $S_{v}$ as given by Equation (10).

(10) $$\{\begin{array}{c} S_{u}^{\prime }=f-\beta \left(I_{u}+I_{v}\right)S_{u}/N\\ S_{v}^{\prime }=g-\beta \left(1-\rho \right)\left(I_{u}+I_{v}\right)S_{v}/N \end{array}$$

This gives us $N^{\prime }=f+g$, for f=f(t) and g=g(t). Integrating both sides gives us $N\left(t\right)=\int_{0}^{t}\left(f\left(s\right)+g\left(s\right)\right)ds.$ If we choose $f\left(t\right)=\left(1-\alpha \right)N_{obj}^{\prime }\left(t\right)$ and $f\left(t\right)=\alpha N_{obj}^{\prime }\left(t\right)$, for a continuous and differentiable function $N_{obj}\left(t\right)$, then $f\left(t\right)+g\left(t\right)=N_{obj}^{\prime }\left(t\right)$ and we expect that $N\left(t\right)=N_{obj}$. The simulation is conducted numerically, which includes the discretization of the time domain, and hence the condition of the differentiability of *N_obj_* is no longer relevant. The subscript objwhich stands for ‘objective’, denotes the preferred dynamics of N(t) that represent a certain school opening scheme. Hence, we can assess the effect of a specific school reopening scheme by choosing the appropriate function $N_{obj}$ that depicts the expected dynamics of the total individuals at any time *t*. Then, we choose three different $N_{obj}\left(t\right)$ values that represent three interesting school reopening schemes:

1. **No school reopening (benchmark)**
  We preserve the size of the population as it was used to generate simulations in the previous section. We set N=4000 for all t>0, which leads to the constant population size for all time. This scenario is a benchmark for the other two scenarios.
2. **Gradual school reopening**
  A gradual school reopening is a scheme that admits students and academical staff gradually until, at some point, the total number of students and staff is reached. In the Institut Teknologi Bandung (ITB), there are approximately 20,000 students and academical staff at any time for a non-pandemic era, which starts with only 4000 individuals in a pandemic era (January until April 2022). Hence, we choose a simple-bounded linearly increasing function $N_{obj}$ as given by:
  $$N_{obj}\left(t\right)=\{\begin{array}{c} 4000\;for\;t<58\;\\ 4000+114\left(t-59\right)\;for\;t\in \left[59,\;200\right]\\ 20,000\;for\;t>201 \end{array}$$
  $t\in {\mathbb{Z}}^{+}$, with t∈[0,58], is the training data interval, which uses N=4000. However, t∈[200,end] represents the total school reopening that starts in September 1, 2022, with N=20,000. The middle period of t∈[59, 200] represents a linearly gradual reopening from 4000 to 20,000. In practice, it is easy to add that f=114(1−α) and g=114α during the period of reopening t∈[59, 200], and f=g=0 otherwise.
3. **Prevalence-tuned school opening**
  The last scenario accommodates the response of the school officials to reduce the school capacity as the disease prevalence level increases. Hence, we assume that the number of N should be related to the number of I. We chose a negative exponential to represent the relation between N and I as follows:
  $$N_{obj}\left(t\right)=\left(20,000-4000\right)e^{-kI\left(t\right)}+4000.$$

This formulation suggests that as I is around zero, then the school officials are about to totally open the school, and N=20,000. The opposite condition with a large number of *I* forces the school restriction and allows only 4000 individuals. This formulation of *N_obj_* is not explicitly time-dependent; instead, it depends on the varying values of I(t). In practice, we can set $f=\left(1-\alpha \right)\left(20,000-4000\right)ke^{-kt}I^{\prime }\left(t\right)$ and $g=\alpha \left(20,000-4000\right)ke^{-kt}I^{\prime }\left(t\right)$.

**Figure 8:**
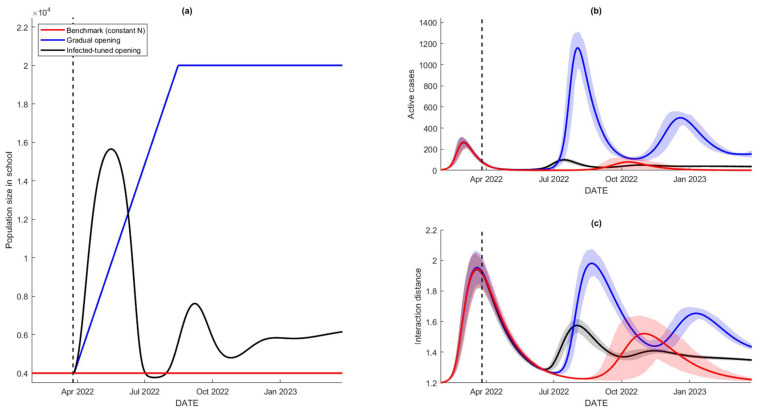
Numerical results of school reopening schemes: no reopening (benchmark) in red, gradual reopening in blue, and case-based reopening in black; (**a**) population sizes for the three scenarios during the school reopening, (**b**,**c**) simulations of the disease prevalence $I=I_{u}+I_{v}$ and the interaction distance *D* for different reopening schemes.

Figure 8 shows the numerical assessment of the three school reopening schemes. The simulations in red are the results that act as a benchmark for the other scenarios. This benchmark scenario gives the constant population size that drives the resurgence of the active cases around October as the average interaction distance increases. However, adding more people into the school through the gradual reopening scheme leads to more infections recorded, which reach a peak around July–August 2022. The surge is expected to happen since we add more people as N increases from 4000 to 20,000 in early September 2022. However, the infection-tuned scheme allows more people to enter the school relative to the other two schemes, yet results in lower cases compared to the second scenario. This is caused by the response of the school officials to reducing the school participants as the cases start to increase. This is the reason why cases increase in the same period as the second scheme but are significantly lower. By this simulation, all scenarios of reopening drive more people to enter the school, leading to more infections. The next section shows how the vaccine-related improvement can solve the problem of reopening without expecting any surge in infections.

#### 5.3.2. Vaccine Coverage and Effectiveness Improvement

**Figure 9:**
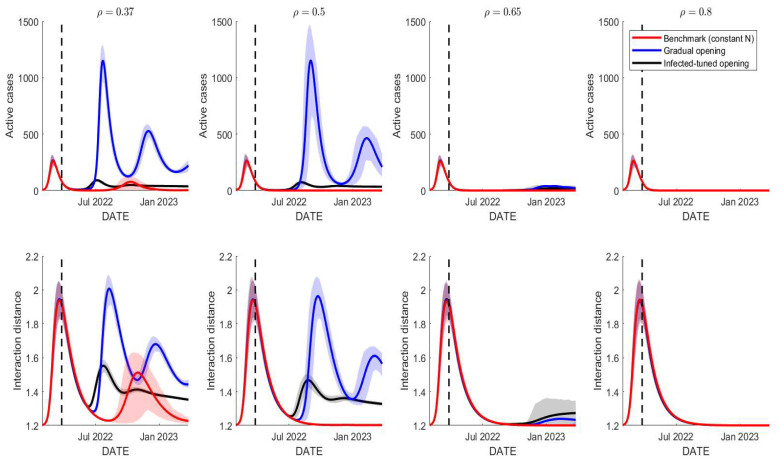
Simulations of the three school reopening schemes under different vaccine efficacies. Improving the vaccine efficacy should reduce the expected numbers of cases. The highest vaccine efficacy in response to the Omicron variant is about 71% [36].

Other than the physical distancing campaign, vaccination is one of the control measures in the spread of COVID-19, especially in a school environment. It has been shown that any school reopening leads to more infections recorded within the society. This section provides a simulation of the three reopening schemes whilst also varying the vaccine efficacy. By April 2022, the current average of vaccine efficacy is around 37%, as most Indonesians have been inoculated twice with SinoVac, which has 37% effectiveness in response to the Omicron variant. The improvement of the vaccine efficacy can be achieved by campaigning for a third vaccine dose with higher efficacy, such as Moderna, Pfizer, or Oxford AstraZeneca. Figure 9 shows the numerical simulations for different values of vaccine efficacy: ρ=37%, 50%, 65%, and 80%.

The figure illustrates the effect of the improvement of the vaccine efficacy, by assuming that 80% of school attendees have received a full-dose vaccine with such efficacy. When the efficacy is improved from 37% to 50%, this affects the first scenario that has only 4000 and cases that are expected to occur in October 2022 vanish. However, ρ=50% is not enough to reduce the other two scenarios (blue and black) significantly as the expected cases remain high for such scenarios. For ρ=65%, most of the expected cases are reduced significantly. Lastly, ρ=80% or higher is expected to reduce a whole surge of cases, at least for the 365-day prediction interval. It can be seen that as the number of cases reduces to zero, the average interaction distance of people approaches its natural level, yet there is no trigger for more infections due to the acquisition of vaccine-induced immunity. These results suggest that any reopening scheme, up to a maximal school capacity of 20,000 individuals, will not lead to any surge in COVID-19 cases as long as 80% of the population has received a vaccine with a minimum 80% efficacy.

## 6. Conclusions

In this study, we provided a modified SIR-type model that incorporates socio-viral behavioral aspects. The first aspect (socio-behavioral) was added to the model by integrating the average interaction distance in society, while the other was added by integrating the critical period in which airborne pathogens remain active. In a general case, a society with a higher resistance rate $\lambda_{1}$ but a lower response rate $\lambda_{2}$ will record more total cases compared to other plausible scenarios. In other words, the mentioned scenario applies to society with people that are hardly accepting new distancing habits and that do not have the awareness of disease prevalence. In contrast, a society with people that easily adapt to new distancing behaviors due to disease transmission, representing a society with a higher $\lambda_{2}$ but a lower $\lambda_{1}$, will result in the least total cases compared to other scenarios. Furthermore, varying the critical period for active airborne pathogens also influence the model behaviors. The higher the critical period, the longer the airborne pathogens actively contribute to the increase in transmission rate.

As a case study, we implemented the proposed data on the spread of COVID-19 in a school environment to preserve the assumption of homogeneity in the population. Using the data on infections, we inferred the unknown parameters using the Bayesian approach. We have shown that the rendered model is well-depicting the training data. Using the inferred parameters, we extrapolated the model and came up with the evidence for a resurgence of cases in around August 2022. The resurgence of the case is purely implied by the return of society to its natural distancing behavior D^* when no new COVID-19 cases are recorded. The dynamics of airborne pathogens load V seem not to influence that significantly due to the short critical period of SARS-CoV-2 to remain active in the air.

In response to the resurgence of cases, we used the model to numerically assess some strategic actions, applicable to the school context, to prevent the resurgence. First, we define some reasonable school reopening schemes that influence the population size: no reopening, gradual reopening, and infection-tuned reopening. While the first has a constant population size N, the second is gradually increasing the population size until it reaches the maximum capacity. Different from the other two, the infection-tuned reopening is a scheme that increases N to its maximum capacity when no diseases are identified, but also allows for decreasing N as the number of cases increases. Though the third scheme seems not practical, an infection-tuned scheme is proven to be the most effective strategy to reopen the school and minimize the risk of the rerise of COVID-19. Second, since we have demonstrated that all school reopening schemes lead to the resurgence of COVID-19 cases, we provide a numerical simulation that justifies the importance of vaccine quality; coverage, and efficacy. With constant vaccination coverage, increasing the vaccine efficacy will reduce the risk of COVID-19 resurgence—a vaccine with an efficacy of more than 80% has been proven to effectively prevent the COVID-19 resurgence, regardless of how society behaves towards the disease spread.

## Figures and Tables

**Table 1:** Average preferred interpersonal distance (in meters) for different types of social relations: strangers, acquaintances, and partners/close relations across all nations. The figure estimations were conducted by Sorokowska et al. [33].

Countries	Social Distance	Personal Distance	Intimate Distance
Romania, Hungary, Saudi Arabia, Turkey, Uganda	1.20–1.40 m	0.90–1.20 m	0.45–0.90 m
Pakistan, Estonia, Colombia, Hong Kong, China, Iran, Malaysia, Czech Republic, Portugal, Kenya, Switzerland, India, Indonesia, Croatia, Ghana, South Korea	1.05–1.20 m	0.75–1.05 m	0.40–0.75 m
Norway, Canada, Nigeria, Brazil, England, Mexico, Poland, Germany, USA, Kazakhstan, Italy, Serbia, Greece, Spain	0.90–1.05 m	0.60–0.75 m	0.40–0.60 m
Russia, Slovakia, Austria, Ukraine, Bulgaria, Peru, Argentina	0.70–0.90 m	0.60–0.70 m	0.30–0.50 m

**Table 2:** Estimated critical periods for SARS-CoV to remain active on several media.

Media	SARS-CoV-2	SARS-CoV-1
Aerosol	10.00 ± 2.00 h	8.00 ± 2.00 h
Copper	11.00 ± 6.00 h	19.00 ± 7.50 h
Cardboard	39.00 ± 9.00 h	8.00 ± 5.00 h
Stainless steel	72.00 ± 15.00 h	50.00 ± 10.00 h
Plastic	90.00 ± 10.00 h	90.00 ± 10.00 h

**Table 3:** Physical distancing campaigns among countries [46,47].

Countries	Physical Distancing (m)
Singapore, United Kingdom, Denmark, France, Hong Kong, China and France	1 m
Australia, Belgium, Greece, Germany, Italy, Spain, Portugal, Switzerland	1.5 m
Canada, United States	2 m

**Table 4:** List of parameters used in evaluating the numerical simulation of System (1) and (2).

Notation	Description	Values
$\gamma_{0}\left(\gamma_{1}\right)$	COVID-19 recovery rate in the case of a lack of healthcare capacity (in the case of excessive healthcare). This parameter governs the time-dependent recovery rate	1/14 (1/6) 1/day
$D^{*}$	Natural interaction distance	1.2 m
$\beta^{*},\;v,$ and w	Intrinsic transmission rate and the contact and airborne transmission adjuster	Calibrated
ρ	Current vaccine efficacy, using SinoVac [29]	0.35
$\lambda_{1}$	The rate of social resistance in the observed community	0.07 1/day
$\lambda_{2}$	The rate of social response in the observed community	0.53 m/day
$\lambda_{3}$	Average concentration of airborne pathogens emitted by one infected individual per day	24 quanta/(day $\mathrm{person}\cdot m^{3})$ [24]
$\lambda_{4}$	Removal rate of airborne pathogens	2 1/day [21]

**Figure A1:**
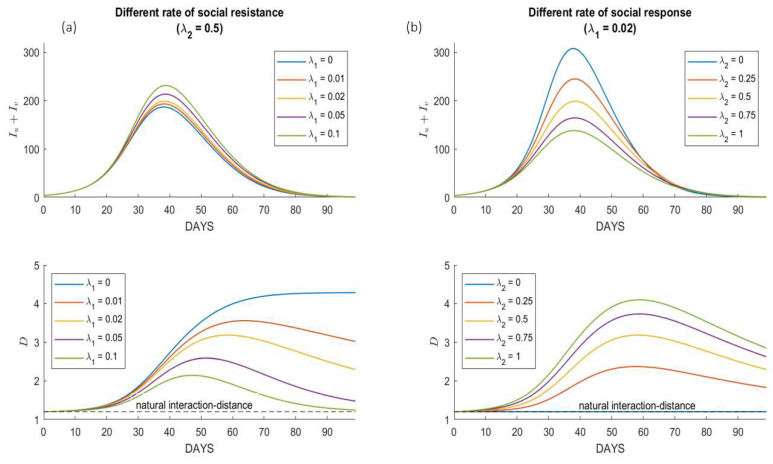
Number of infected individuals in society of population 1000 individuals under different values of socio-resistance and social response rates. (**a**) five different values of socio-resistance by setting $\lambda_{2}=0.5$. Each scenario has ${ℛ}_{0}=$, for ascending order of $\lambda_{1}$. (**b**) Five different values of social response by setting $\lambda_{1}=0.02$.

**Figure A2:**
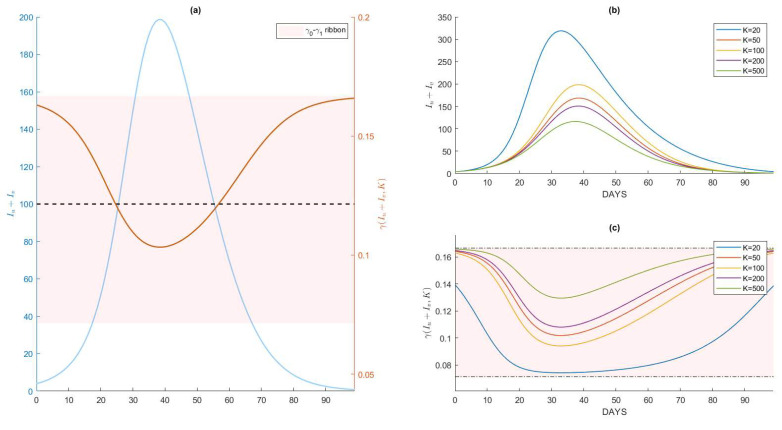
Numerical simulations under different values of healthcare capacities: (**a**) comparison between the dynamics of active cases and the rate of recovery using the same parameters as used in Figure A1, (**b**,**c**) sensitivity analysis of $I_{u}+I_{v}$ and the rate of recovery γ under different value of healthcare capacity K.

## Appendix A. Model Analysis and Threshold Number

For the sake of simplicity, we drop the vaccination effect and hence merge all compartments having indices u and v together. To generate both the disease-free and endemic equilibria, we added the natural disease in all state compartment (SIR), with μ represents the natural death rate. By substituting β(D, V) and γ(I, K) with those given by Equations (3) and (4), we have our system be rewritten as follows.

$$\{\begin{array}{c} S^{\prime }=-\beta^{*}{\left(\frac{2D^{*}}{D^{*}+D}\right)}^{v}{\left(\frac{V+V^{*}}{V^{*}}\right)}^{w}\frac{SI}{N}\;\\ I^{\prime }=\beta^{*}{\left(\frac{2D^{*}}{D^{*}+D}\right)}^{v}{\left(\frac{V+V^{*}}{V^{*}}\right)}^{w}\frac{SI}{N}-\left(\gamma_{0}+\left(\gamma_{1}-\gamma_{0}\right)\frac{K}{I+K}\right)I\\ \begin{array}{c} R^{\prime }=\left(\gamma_{0}+\left(\gamma_{1}-\gamma_{0}\right)\frac{K}{I+K}\right)I\\ \begin{array}{c} D^{\prime }=-\lambda_{1}\left(D-D^{*}\right)+\lambda_{2}\left(I/N\right)\\ V^{\prime }=\lambda_{3}I-\lambda_{4}V \end{array} \end{array} \end{array}$$

All plausible equilibria of System (5) are obtained by solving this nonlinear system, that is modified by adding the recruitment and natural death rate in order to get the Endemic Equilibrium.

$$0=A-\beta^{*}{\left(\frac{2D^{*}}{D^{*}+D}\right)}^{v}{\left(\frac{V+V^{*}}{V^{*}}\right)}^{w}\frac{SI}{N}-\mu S$$

$$0=\beta^{*}{\left(\frac{2D^{*}}{D^{*}+D}\right)}^{v}{\left(\frac{V+V^{*}}{V^{*}}\right)}^{w}\frac{SI}{N}-\left(\gamma_{0}+\left(\gamma_{1}-\gamma_{0}\right)\frac{K}{I+K}\right)I-\mu I$$

$$0=\left(\gamma_{0}+\left(\gamma_{1}-\gamma_{0}\right)\frac{K}{I+K}\right)I$$

$$0=-\lambda_{1}\left(D-D^{*}\right)+\lambda_{2}\left(I/N\right)$$

$$0=\lambda_{3}I-\lambda_{4}V$$

The disease-free equilibrium (DFE) can be obtained by plugging $\hat{I}=0$ to the system, which leads to $DFE=\left\{S^{*},0,R^{*},D^{*},0\right\}$. Using the next generation matrix method, the formula of the basic reproductive ratio is given by

$$R_{0}=\frac{\beta^{*}}{\gamma +\mu }$$

with the term of ${\left(\frac{2D^{*}}{D^{*}+D}\right)}^{v}{\left(\frac{V+V^{*}}{V^{*}}\right)}^{w}$ vanishes to 1 as all states approach the DFE. This quantity will determine whether the state will approach the DFE (when $R_{0}<1$) or otherwise approach the other equilibrium point, namely the EE.

## Appendix B. Numerical Sensitivity Analysis of the Socio-Behavioral Parameters

In this study, the behaviors of society that being accommodated by the model is the rate of social resistance and social response. Socio-resistance rate, denoted with $\lambda_{1}$, represents the resistance of society for distancing their interaction due to the figure prevalence when I is not significantly zero. When the figure prevalence is close to zero, the resistance rate depicts how fast the society to live back with their natural interaction-distance $D^{*}$. In contrast, the rate of society response depicts the increase in interaction distancing per increase in point prevalence, which inhibits the disease spread when this value is set high. In this section, we provide the figure of infected individuals (per one thousand of population) as $\lambda_{1}$ and $\lambda_{2}$ is set varied.

Given in Figure A1, the setting of $\lambda_{1}=0$ while $\lambda_{2}=0.5$ depicts the situation that people in society tend to response to the figure prevalence by distancing interaction but have no intention to return on their previous interaction habit. The blue line in the bottom-left corner represents the average interaction-distance in the mentioned scenario that levels off in D=4.2 without approaching the natural interaction-distance. By fixing $\lambda_{2}=0.5$, the increment of $\lambda_{1}$ will lower the average interaction-distance and fasten the dynamics of D to approach $D^{*}$. Poor society in certain regions tend to hasten on returning back on their previous habits (to work, study, etc) once the decrease in the point prevalence are declared, which in this model is considered to have relatively higher $\lambda_{1}$. In contrast, some regions in developed countries may have lower value of $\lambda_{1}$. Hence, the model requires the homogeneity of society; the smaller the society, the easier to assume, such as: students and academic staff in closed school, workers.

Right-hand side figures in Figure A1 depicts how social response to point prevalence affects the dynamics of both infected individuals and interaction-distance. The scenario setting *λ*_1_ = 0 portrays the society with no attention to current disease spread, implying to a steady interaction-distance to its natural habit. The higher the setting of $\lambda_{2}$, the more responsive the people in society to the current point prevalence.

## Appendix D. Bayesian Hierarchical for Parameters’ Estimation

There are three parameters that are estimated by the integration of the provided data $D_{\alpha }$. We assume that those three parameters are each a realization of a complete posterior distribution. Let us assume that $D_{\alpha }\left(i\right),i=1,2,\ldots ,n$ denotes the daily cases of COVID-19 at day i, while $f\left(i;\hat{\theta }\right)$ represents the dynamics of the daily cases evaluated from the proposed model given that $\hat{\theta }={\left[\beta^{*},v,w\right]}^{T}$. We expect that observation $D_{\alpha }\left(i\right)$ (experimental data or observed data) to be equal as the model response $f\left(i,\hat{\theta }\right)$ plus the independent and identically distributed error $\varepsilon_{i}$, with mean zero and variance $\sigma^{2}$. Hence, we can write:

$$D_{\alpha }\left(i\right)=f\left(i;\hat{\theta }\right)+\varepsilon_{i}$$

with $\varepsilon_{i}\;N\left(0,\sigma^{2}\;\right)$. The goal is to estimate the posteriod distribution of $\pi \left(\hat{\theta }|D_{\alpha }\left(i\right)\right)$, which quantify the probability of parameter $\hat{\theta }$ given the set of observational data.

$$\pi \left(\hat{\theta }|D_{\alpha }\left(i\right)\right)=\frac{ℒ(D_{\alpha }\left(i\right)|\hat{\theta })\pi_{0}\left(\hat{\theta }\right)}{NL}$$

with $ℒ(D_{\alpha }\left(i\right)|\left(\hat{\theta }\right))$ represent the likelihood and $\pi_{0}\left(\hat{\theta }\right)$ is the prior knowledge of the estimated parameters. In terms of the data fitting, it is common to define the likelihood function as given in the following formula:

$$(D_{\alpha }\left(i\right)|\left(\hat{\theta }\right))=exp\left(-\frac{SS_{q}}{Var}\right)$$

with $SS_{s}=\sum_{i}^{n}{\left(D_{\alpha }\left(i\right)-f\left(i;\hat{\theta }\right)\right)}^{2}$. We will use the MCMC method (with a Metropolis-Hastings Algorithm) to get samples from the posterior distributions given in 860. Since we have no prior knowledge for each of the estimated parameters, we choose the prior of normal distribution with zero mean, but high in variance. In MATLAB, we used the built-in function provided by Grinsted, A. available in https://www.mathworks.com/matlabcentral/fileexchange/49820-ensemble-mcmc-sampler (accessed on 17 June 2022).
